# Self-care behaviors and glycemic control in Nigerian patients with type 2 diabetes: a pilot cross-sectional study

**DOI:** 10.1186/s41256-025-00427-9

**Published:** 2025-11-11

**Authors:** Muhammad Idris Abdullahi, Yanxin Bi, Minmin Wang, Mohammad Hamza Ashiru, Zhi-Jie Zheng, Yinzi Jin

**Affiliations:** 1 Department of Global Health, School of Public Health, Peking University, Beijing, China; 2 Department of Physiotherapy, Federal Medical Center Gusau, Zamfara State, Nigeria; 3 Department of Physiotherapy, Faculty of Basic Medical Sciences, Zamfara State University, Talata Mafara, Zamfara State Nigeria; 4 Department of Public Health, Godiya Disability Inclusion and Development Initiative (GDID), Dutse, Jigawa State Nigeria; 5 Institute for Global Health and Development, Peking University, Beijing, China

**Keywords:** Self-care behaviors, Glycemic control, Type 2 diabetes management, Nigeria, Pilot cross-sectional study

## Abstract

**Background:**

Self-care behaviors are essential for managing type 2 diabetes mellitus (T2DM), even among patients receiving specialized clinical care. However, limited evidence exists on how these behaviors affect glycemic outcomes in Nigerian patients already engaged with endocrinology services. This study assessed self-care practices and their association with glycemic control among T2DM patients attending two specialized clinics in Gusau, Nigeria.

**Methods:**

A cross-sectional study was conducted among 262 adult T2DM patients from two endocrinology clinics in Gusau, Nigeria. Participants were recruited using a convenience sampling approach during routine clinic visits. Each completed questionnaires on demographics, the Summary of Diabetes Self-Care Activities (SDSCA), and the Morisky Medication Adherence Scale (MMAS-4). Fasting blood glucose levels were used to assess glycemic control. Logistic regression analyzed the association between self-care behaviors and glycemic outcomes; linear regression identified influencing factors.

**Results:**

Among 262 participants, 45.8% had uncontrolled fasting blood glucose (≥ 7 mmol/L). Higher overall self-care scores were significantly associated with better glycemic control (OR 0.83; 95% CI 0.78–0.88; *P* < 0.001). Dietary adherence (OR 0.72; 95% CI 0.61–0.85), physical activity (OR 0.74; 95% CI 0.65–0.84), and blood glucose monitoring (OR 0.22; 95% CI 0.09–0.54) were significantly associated with improved glycemic outcomes. Foot care (OR 0.98; *P* = 0.797) and medication adherence (OR 3.21; *P* = 0.095) showed no significant association. Males, older adults (≥ 60 years), and Yoruba/Igbo participants had lower dietary scores, while exercise scores were higher among males, singles, and Igbo patients. Longer diabetes duration (≥ 20 years) was linked to better medication adherence and exercise.

**Conclusions:**

Self-care behaviors were significantly associated with glycemic control among T2DM patients attending two specialized endocrinology clinics in Gusau, Nigeria. Interventions tailored to demographic and cultural contexts are essential to strengthen adherence in key areas such as diet, physical activity, and glucose monitoring. These findings underscore the importance of supporting self-management, even in patients with established access to clinical care.

**Electronic supplementary material:**

The online version contains supplementary material available at 10.1186/s41256-025-00427-9.

## Introduction

As indicated by the International Diabetes Federation, the number of adults living with diabetes reached 537 million in 2021, and this figure is projected to rise to 783 million by 2045 if immediate interventions are not implemented [1]. The burden of diabetes is especially severe in low- and middle-income countries (LMICs), with over 341 million individuals at risk of developing type 2 diabetes mellitus (T2DM) [2]. Sub-Saharan Africa, including Nigeria, has seen a significant increase in diabetes prevalence, particularly in urban areas where rapid lifestyle changes, such as reduced physical activity and poor dietary habits, exacerbate the problem [3, 4]. Nigeria, with a population of over 193 million, bears the second-highest diabetes burden in Africa, with an estimated 2.7 million individuals affected [5]. In addition to these challenges, diabetes in this region frequently interacts with infectious diseases such as tuberculosis and HIV/AIDS, which further complicates management efforts [6, 7].

The management of T2DM is multifaceted, requiring both pharmacological and non-pharmacological strategies to achieve effective glycemic control and minimize complications. However, in Nigeria, the healthcare system faces considerable challenges, especially in rural, low-resource settings. Limited access to healthcare services, a lack of comprehensive insurance coverage, and a heavy reliance on out-of-pocket payments hinder patients’ ability to manage their condition effectively [5, 8]. While pharmacological interventions such as insulin and oral glucose-lowering drugs are available, financial constraints and poor accessibility often impede their use [8–10]. Furthermore, non-pharmacological approaches, especially self-care behaviors, including adherence to dietary plans, physical activity, and blood glucose monitoring, are vital for managing diabetes independently, as patients spend significantly more time outside healthcare facilities [10, 11]. Despite the recognized importance of self-care practices, research is limited on the prevalence and determinants of these behaviors among Nigerians with T2DM, particularly in underserved and lower socioeconomic populations [12, 13].

Self-care behaviors are fundamental to effective diabetes management, enabling patients to actively engage in their care and mitigate the risk of complications [14]. Despite their recognized importance, there is a paucity of research investigating self-care practices among individuals with T2DM in Nigeria. Existing studies predominantly focus on urban populations and individuals from higher socioeconomic groups, but key aspects of self-care behaviors remain underexplored operate even among patients with established access to specialized diabetes care. Gusau was selected as the study setting due to its position as the capital of Zamfara State, where the selected clinics serve as referral centers for diabetes care across the region. This study aims to characterize self-care behaviors and their association with glycemic control among T2DM patients attending two specialized endocrinology clinics in Gusau, Nigeria, with a focus on identifying modifiable factors within this clinically engaged population. Specifically, the study seeks to identify determinants of self-care behaviors, quantify the prevalence of these practices, and analyze their correlations with blood glucose levels in this demographic. By focusing on patients receiving specialized clinical care, this study addresses an important gap in understanding self-care behaviors in healthcare settings where treatment is available but adherence challenges persist. The findings may help inform strategies to enhance diabetes management support in similar clinical contexts across sub-Saharan Africa.

## Methods

### Design, setting, and participants

This cross-sectional study was conducted in the Endocrinology Clinic of the Federal Medical Center and Dr. Ahmad Sani Yariman Bakura Specialist Hospital in Gusau Town, Nigeria, between August 2023 and March 2024. These clinics were selected to examine self-care behaviors in patients with established access to specialized diabetes care. These hospitals were selected due to their large patient volumes and role as major endocrinology referral centers in Zamfara State. The research participants included adult patients with T2DM actively receiving clinical management at these facilities. Patients were excluded if they were pregnant, severely ill, or unable to provide informed consent to participate. This sampling approach intentionally focused on patients engaged in clinical care, excluding those without access to specialized diabetes services. Given operational constraints such as limited clinic hours and staffing, a convenience sampling strategy was employed to generate preliminary data on self-care behaviors in a clinical setting. This approach was deemed necessary as a first step to designing targeted interventions in similar contexts, where the goal is to improve the self-care practices of patients with existing access to specialized care. Based on statistical calculations (α = 0.05, *P* = 0.12, Z_α/2_ = 1.96, and d = 0.05), the minimum sample size required was 162, which was increased to 203 to account for a 25% non-response rate. Participants were recruited using a convenience sampling strategy. Specifically, all eligible patients who attended the clinics during the study period and met the inclusion criteria were approached consecutively and invited to participate. Informed consent was obtained from all participants, and the study received ethical approval. Participation rates were higher than anticipated, and a total of 262 diabetic patients were included in the study. These hospitals were selected due to their large patient volumes and role as major endocrinology referral centers in Zamfara State.

### Instruments for data collection

Data on self-care behaviors were collected using the Summary of Diabetes Self-Care Activities (SDSCA) questionnaire and the Morisky Medication Adherence Scale-4 (MMAS-4). Both instruments are widely recognized and validated tools used in numerous studies to assess self-care behaviors and medication adherence among individuals with diabetes. These instruments are publicly available and have been extensively utilized in diabetes-related research [15, 16]. The SDSCA questionnaire, a validated tool, assesses self-care behaviors related to diet, physical exercise, blood glucose testing, and foot care [15]. Participants reported the number of days they engaged in each activity over the past week, and scores were calculated for each dimension by summing the relevant item scores, with reverse scoring for the fourth dietary item. The MMAS-4 scale assesses medication adherence through four questions, each with a 'Yes' or 'No' response. A 'Yes' answer is scored 0, while a 'No' answer is scored 1. The total score is the sum of the individual item scores, ranging from 0 to 4. A higher total score indicates better medication adherence [15–17].

The total score for the SDSCA is the sum from 1 to 10 items, with the fourth item being scored in reverse. The subscales were scored as follows: Diet = total number of days for items 1 to 4 (item 4 reverse scoring); Exercise = total number of days for items 5 and 6; Blood glucose testing = total number of days for items 7 and 8; Foot care = total number of days for items 9 and 10. The total score for the MMAS-4 was calculated by summing the scores for the four items, and medication adherence was treated as a continuous variable in all subsequent analyses. Subsequently, the total scores for the SDSCA and MMAS-4 were added together. Although the SDSCA and MMAS-4 assess distinct domains, the total score was used descriptively to provide an overall summary of self-care engagement; individual subscale scores were retained for analytical comparisons.

### Data collection

Patients diagnosed with T2DM who met the eligibility criteria and provided informed consent were administered sociodemographic questionnaires, the SDSCA scale, and the MMAS-4. Professional nurses measured blood pressure, blood glucose levels, and BMI. The data collection process took place over a period of 3 months from August 2023 to October 2023. Each research assistant was trained using a standardized protocol to ensure consistency in questionnaire administration and clinical measurements. For patients with impaired vision, the principal investigator read the questionnaire aloud and accurately recorded their responses. To ensure data quality, field-level checks were performed regularly, and any discrepancies in the data were corrected before finalizing the dataset. Participant anonymity was maintained throughout the data collection process.

### Data analysis

We described the demographic and clinical characteristics of the study participants, as well as their responses to the questionnaire items. In accordance with the World Health Organization (WHO) diagnostic guidelines, a fasting blood glucose level of 126 mg/dL (7 mmol/L) or higher on two separate occasions is used for diagnosing diabetes [18]. For glycemic control, we defined controlled T2DM as a fasting plasma glucose level ≤ 130 mg/dL or an HbA1c ≤ 7%, in line with the American Diabetes Association (ADA) guidelines. Group comparisons were conducted using independent samples *t*-tests for continuous variables, analysis of variance (ANOVA) for comparisons across multiple groups, and chi-square tests for categorical variables. Multivariate logistic regression was used to assess the association between self-care behaviors and glycemic control, while multivariate linear regression identified factors influencing self-care behaviors. Diagnostic tests were performed to validate the assumptions of the regression models. For the multivariate linear regression models, adjusted *R*^*2*^, F-statistics, and Root Mean Square Error (RMSE) were reported to evaluate the explanatory power and fit of the models. Multicollinearity was assessed using the Variance Inflation Factor (VIF), with a threshold of 10 used to identify potential issues. Residual normality was evaluated using the Shapiro–Wilk test. While deviations from normality were observed, the large sample size justifies the robustness of the regression results.All statistical analyses were conducted using Stata MP (Version 18.0; Stata Corp LLC, TX, USA), with a significance level set at *P* < 0.05 (two-side).

## Results

### Socio-demographic and clinical characteristics of the diabetic participants

A total of 262 diabetic patients actively receiving specialized endocrinology care participated in this study, with 53.44% being female. The largest age group was 41–60 years (50.38%), followed by > 60 years (34.73%). Most participants were married (58.02%), of Hausa ethnicity (71.37%), and Muslim (97.33%). Blood glucose control was achieved in only 54.20% of participants, with 45.80% having elevated fasting blood glucose levels (≥ 7 mmol/L). Nearly 74.81% of participants had normal blood pressure, and 54.20% had a normal BMI (Table 1)**.** Differences in fasting blood glucose levels across socio-demographic variables indicated significant associations with age, marital status, duration of illness, and ethnicity, but not with gender, BMI, or blood pressure. (Supplementary Table 1).

**Table 1:** Socio-demographic and clinical characteristics of the patients

Variables	Participants (N = 262)	Percentage (%)
Gender		
Female	140	53.4
Male	122	46.6
Age group (years)		
18–40	39	14.9
41–60	132	50.4
> 60	91	34.7
Marital status		
Married	152	58.0
Single	110	42.0
Ethnicity		
Hausa	187	71.4
Yoruba	48	18.3
Igbo	27	10.3
Religion		
Islam	255	97.3
Christianity	7	2.7
Duration of illness (years)		
1–10	172	65.7
11–20	70	26.7
> 20	20	7.6
Fasting blood glucose test (mmol/l)		
Normal (< 7 mmol/L)	142	54.2
Elevated (≥ 7 mmol/L)	120	45.8
Blood pressure (mmHg)		
Normal (< 130/80 mmHg)	196	74.8
High Blood Pressure (≥ 130/80 mmHg)	66	25.2
Body Mass Index, BMI (kg/m^2^)		
Under weight (< 18.5)	18	6.9
Normal (18.5–24.9)	142	54.2
Overweight (25.0–29.9)	42	16.0
Obese (≥ 30)	60	22.9

### Self-care behaviors for diabetes

The mean total self-care score was 41.65 ± 12.33, comprising behaviors related to diet, exercise, blood glucose monitoring, foot care, and medication adherence **(**Table 2**)**. Foot care had the highest adherence (11.54 ± 5.33), while daily blood glucose testing had the lowest (1.25 ± 2.80). Supplementary Table 2 showed the total score and differences in scores for the total and each dimension of self-care behaviors among patients with different socio-demographic and clinical characteristics. For the self-care behaviors, the differences in total scores among marital status, blood pressure, ethnicity, and blood glucose levels were significant (*P* < 0.05). The score differences in more dimensions are detailed in Supplementary Table 2.

**Table 2:** Summary items of diabetes self-care activities

Items	Mean (Min, Max)	SD
SDSCA Scale	38.08 (10, 63)	12.17
Diet	19.67 (7, 28)	6.97
Exercise	5.61 (0, 14)	5.28
Self-daily blood glucose testing behaviors	1.25 (0, 14)	2.80
Foot care	11.54 (0, 14)	5.33
MMAS-4 Scale (Medication adherence)	3.58 (2, 4)	0.59
Total score	41.65 (13, 67)	12.33

SDSCA: Summary of Diabetes Self-Care Activities; MMAS-4: Morisky’s Medication Adherence Scale-4

### Relationship between self-care behaviors and blood glucose test levels

Multivariate logistic regression analysis (Supplementary Table 3) indicated that higher total self-care scores were significantly correlated with improved glycemic control (OR 0.83, *P* < 0.001), suggesting that for each unit increase in self-care score, patients were 17% less likely to have uncontrolled blood glucose levels. Among the self-care behaviors dimensions, dietary habits (OR 0.72, *P* < 0.001) were strongly associated with better control, implying a 28% reduced likelihood of poor glycemic control with improved dietary adherence. Similarly, exercise (OR 0.74, *P* < 0.001), and daily blood glucose testing (OR 0.22, *P* = 0.001) were all significantly linked to better blood glucose control levels. Conversely, foot care (OR 0.98, *P* = 0.797) and medication adherence (OR 3.21, *P* = 0.095) did not show a significant association with blood glucose levels (Table 3).

**Table 3:** Association between five dimensions of self-care behaviors and blood glucose levels

Variables	OR	SE	Z	*P*	95% CI	
					Lower	Upper
Diet	0.72	0.06	− 3.90	< 0.001	0.61	0.85
Exercise	0.74	0.05	− 4.54	< 0.001	0.65	0.84
Self-daily blood glucose testing behaviors	0.22	0.10	− 3.26	0.001	0.09	0.54
Foot care	0.98	0.06	− 0.26	0.797	0.86	1.12
Medication adherence	3.21	2.25	1.67	0.095	0.82	12.66

The model has adjusted for body mass index, blood pressure, gender, age group, marital status, ethnicity, and duration of illness

### Factors influencing self-care behaviors in diabetic patients

Multivariate regression analysis revealed that, compared to females, male patients had lower scores in dietary self-management (*β* = − 0.19, *P* < 0.01), foot care (*β* = − 0.13, *P* < 0.05), and medication adherence (*β* = − 0.17, *P* < 0.01), but higher scores in exercise self-management (*β* = 0.23, *P* < 0.001). Patients aged ≥ 60 years had reduced dietary self-management scores (*β* = − 0.19, *P* < 0.05), while those aged 41–60 years had increased scores for blood glucose monitoring (*β* = − 0.27, *P* < 0.01) and decreased scores for foot care (*β* = − 0.23, *P* < 0.01), relative to the youngest group. Compared with Hausa participants, Igbo patients had higher exercise scores (*β* = 0.19, *P* < 0.01), but lower scores in dietary management (*β* = − 0.13, *P* < 0.05), blood glucose monitoring (*β* = − 0.16, *P* < 0.01), foot care (β = − 0.49, *P* < 0.001), and medication adherence (*β* = − 0.43, *P* < 0.001). Single individuals, compared to married ones, showed higher scores in dietary (*β* = 0.15, *P* < 0.05) and exercise behaviors (*β* = 0.27, *P* < 0.001), but lower scores in blood glucose monitoring (*β* = − 0.18, *P* < 0.01). A diabetes duration of more than 20 years was associated with higher scores in exercise (*β* = 0.13, *P* < 0.01) and medication adherence (*β* = 0.15, *P* < 0.05), and lower scores in foot care (*β* = − 0.19, *P* < 0.001), compared to those with shorter disease duration (Table 4 and Supplementary Table 4).

**Table 4:** Analysis of factors influencing the five dimensions of self-care in diabetic patients

Variables	Diet	Exercise	Self daily blood glucose testing behaviors	Foot care	Medication adherence
Body Mass Index, BMI (kg/m^2^)					
Under weight					
Normal	− 0.09 (− 4.96, 2.47)	0.04 (− 2.50, 3.36)	− 0.25 (− 2.94, 0.10)	0.06 (− 1.79, 3.19)	− 0.07 (− 0.38, 0.21)
Overweight	− 0.02 (− 4.28, 3.40)	− 0.02 (− 3.30, 2.75)	− 0.25^*^ (− 3.19, − 0.04)	0.06 (− 1.88, 3.27)	0.02 (− 0.28, 0.33)
Obese	− 0.09 (− 5.17, 2.44)	− 0.14 (− 4.58, 1.43)	− 0.25 (− 3.06, 0.05)	0.04 (− 2.05, 3.05)	− 0.01 (− 0.32, 0.28)
Blood pressure (mmHg)					
Normal					
High	− 0.06 (− 2.94, 0.95)	0.06 (− 0.83, 2.24)	0.08 (− 0.26, 1.33)	0.31^***^ (2.48, 5.08)	− 0.14^*^ (− 0.35, − 0.04)
Gender					
Female					
Male	− 0.19^**^ (− 4.39, − 0.97)	0.23^***^ (1.05, 3.75)	0.12 (− 0.03, 1.37)	− 0.13^*^ (− 2.56, − 0.27)	− 0.17^**^ (− 0.34, − 0.07)
Age group (years)					
18–40					
41–60	− 0.16 (− 4.67, 0.33)	0.14 (− 0.50, 3.44)	0.27^**^ (0.48, 2.52)	− 0.23^**^ (− 4.11, − 0.76)	0.10 (− 0.08, 0.31)
> 60	− 0.19^*^ (− 5.35, − 0.26)	− 0.09 (− 3.05, 0.96)	0.01 (− 0.94, 1.15)	0.04 (− 1.22, 2.20)	0.05 (− 0.13, 0.27)
Marital status					
Married					
Single	0.15^*^ (0.26, 3.93)	0.27^***^ (1.49, 4.38)	− 0.18^**^ (− 1.79, − 0.29)	− 0.11 (− 2.39, 0.07)	0.03 (− 0.11, 0.18)
Ethnicity					
Hausa					
Yoruba	− 0.16^**^ (− 5.06, − 0.80)	− 0.03 (− 2.05, 1.31)	− 0.04 (− 1.18, 0.56)	− 0.02 (− 1.79, 1.07)	− 0.15^**^ (− 0.40, − 0.06)
Igbo	− 0.13^*^ (− 5.52, − 0.22)	0.19^**^ (1.14, 5.32)	− 0.16^**^ (− 2.58, − 0.41)	− 0.49^***^ (− 10.30, − 6.75)	− 0.43^***^ (− 1.03, − 0.61)
Duration of illness (years)					
1–10					
11–20	− 0.21^***^ (− 5.10, − 1.47)	− 0.05 (− 2.04, 0.82)	− 0.04 (− 0.98, 0.51)	0.25^***^ (1.82, 4.25)	− 0.10 (− 0.27, 0.01)
> 20	0.01 (− 3.13, 3.42)	0.13^*^ (0.02, 5.19)	0.08 (− 0.50, 2.18)	− 0.19^***^ (− 6.10, − 1.71)	0.15^*^ (0.07, 0.58)

^*^P < 0.05, ^**^P < 0.010, ^***^P < 0.001

## Discussion

This study aimed to assess self-care behaviors among Nigerian T2DM patients receiving specialized endocrinology care and to identify the socio-demographic and clinical factors influencing these behaviors. We examined five key behavioral dimensions: diet, physical activity, blood glucose monitoring, foot care, and medication adherence, and investigated their relationships with glycemic control. Our findings provide important insights into the patterns of self-care behaviors and their determinants in this population, with significant implications for clinical practice and health policy in Nigeria and similar settings. These insights should be interpreted in context. Our participants were patients actively engaged with specialized endocrinology services, who may differ from the broader T2DM population in access, education, and engagement.

The most satisfactory behaviors observed were foot examination and medication adherence, with 82.44% of participants reporting that they inspect their feet and 62.60% stating that they strictly adhere to their medications. These findings are similar to those reported in other studies [19, 20]. The high foot care adherence may be partially attributed to cultural and religious practices, as 97.33% of participants identified as Muslim and 71.37% were of Hausa ethnicity, where regular foot washing is a religious practice. However, 73.28% and 70.61% did not perform self-monitoring of blood glucose in accordance with the recommendations of their healthcare providers over the past seven days and adhered to the recommended frequency as advised by their healthcare providers. Thus, the participants demonstrated suboptimal glycemic control. This is analogous to a prior study that similarly observed unsatisfactory and poor glycemic control among their study participants, which they reported to be potentially associated with noncompliance with self-care behaviors [21]. In alignment with our study findings and those of previous research, these investigations also demonstrated that the majority of participants in the study reported inadequate adherence to diabetic self-care practices, occurring less than four days a week [22]. Noncompliance with self-care is presumed to cause suboptimal health outcomes, diabetic complications, death, or, subsequently, increased healthcare costs [23].

Our regression analysis confirmed significant associations between certain self-care behaviors and glycemic control. Dietary habits (OR 0.72, *P* < 0.001), physical activity (OR 0.74, *P* < 0.001), and blood glucose monitoring (OR 0.22, *P* = 0.001) were significantly associated with improved glucose control, aligning with established literature on diabetes management [24, 25]. Specifically, dietary practices significantly influenced glycemic control, reinforcing the importance of nutritional counseling that addresses cultural dietary patterns in Nigerian populations [26–28]. Similarly, physical activity improves insulin sensitivity and helps lower blood glucose levels [29], while regular glucose monitoring enables timely treatment adjustments [30], forming a foundation for effective diabetes management.

Several sociodemographic and clinical factors influenced specific self-care behaviors. Male gender was associated with poorer dietary management (*β* = − 0.19, *P* < 0.01), lower foot care (*β* = − 0.13, *P* < 0.05), and reduced medication adherence (*β* = − 0.17, *P* < 0.01), but better exercise practices (*β* = 0.23, *P* < 0.001). This gender disparity suggests the need for tailored interventions addressing male-specific barriers to adherence [31, 32]. Age also emerged as a significant factor, with adults over 60 showing poorer dietary management (*β* = − 0.19, *P* < 0.05), potentially due to financial constraints or declining health [33].

Notably, Igbo participants showed distinct self-care patterns compared to the predominantly Hausa sample, including poorer dietary management (*β* = − 0.13, *P* < 0.05), lower blood glucose testing (*β* = − 0.16, *P* < 0.01), poorer foot care (*β* = − 0.49, *P* < 0.001), and reduced medication adherence (*β* = − 0.43, *P* < 0.001), but better exercise practices (*β* = 0.19, *P* < 0.01). These variations may reflect cultural, social, or structural factors and highlight the need for further investigation into how ethnicity influences diabetes care [34].

Marital status influenced self-care behaviors, with single individuals demonstrating better dietary management (*β* = 0.15, *P* < 0.05) and higher exercise adherence (*β* = 0.27, *P* < 0.001), but poorer blood glucose monitoring (*β* = − 0.18, *P* < 0.01). This may reflect greater flexibility in meal planning and physical activity for single individuals but potential challenges in maintaining consistent monitoring routines [35, 36].

Our findings revealed a complex relationship between diabetes duration and self-care behaviors. Patients with longer disease duration (> 20 years) demonstrated better medication adherence (*β* = 0.15, *P* < 0.05) and exercise practices (*β* = 0.13, *P* < 0.01), suggesting that long-term experience with the disease may foster better understanding of treatment importance and develop routines that support these aspects of self-management [37, 38]. However, the same group showed poorer foot care practices (*β* = − 0.19, *P* < 0.001), while those with 11–20 years duration exhibited poorer dietary management (*β* = − 0.21, *P* < 0.001). This indicates that duration-related self-care patterns are not uniform across all behavioral dimensions. The improved medication adherence among long-term patients aligns with previous research suggesting that extended experience with diabetes management can lead to better integration of medication routines into daily life [39]. Conversely, the decline in certain self-care practices with extended duration might reflect diabetes burnout, where the psychological burden of long-term disease management leads to reduced adherence in more demanding behavioral domains [40]. Diabetes burnout has been increasingly recognized as a critical psychological barrier to sustained self-management, associated with emotional exhaustion, detachment from care routines, and increased risk of complications [41]. Addressing burnout through ongoing mental health support and routine screening may be essential for preserving long-term adherence and quality of life in individuals with chronic diabetes. These findings highlight the need for targeted refresher education and psychological support for long-term diabetes patients, focusing on maintaining comprehensive self-care practices beyond medication adherence.

It is important to interpret these findings within the context of our study population, which comprised patients actively receiving care from specialized endocrinology clinics. The relatively high self-reported medication adherence (mean score = 3.58) suggests generally good adherence behavior in this clinically engaged group. However, medication adherence was not significantly associated with glycemic control in our regression analysis (*P* = 0.095), indicating that its direct impact on blood glucose levels could not be established. This may reflect selection bias: regular clinic attendees are typically more health-conscious, better educated, and more motivated to engage in self-care. Such characteristics may partly explain the favorable adherence patterns observed in this sample. Moreover, the predominance of Hausa ethnicity (71.37%) limits the generalizability of our findings to Nigeria’s broader and more ethnically diverse population.

For practice and policy implications, our findings suggest several important directions that may be organized into patient-level and system-level interventions. Patient-level interventions include culturally-sensitive educational programs targeting specific demographic groups, psychological support to address long-term disease fatigue, especially among patients with extended diabetes duration. Strengthening community-based support systems can also help promote daily self-care behaviors. On the system level, efforts should focus on subsidizing glucose monitoring supplies to reduce financial barriers, particularly for older adults and individuals from lower socioeconomic backgrounds, expanding access to specialized endocrinology services for underserved populations, and strengthening healthcare provider training in culturally competent communication to address beliefs and practices that influence self-care behaviors.

This study provides valuable insights into self-care behaviors among individuals with type 2 diabetes mellitus in Nigeria; however, it has several limitations. First, as the study was conducted in two specialized endocrinology clinics in a single urban area of Zamfara State, the findings may not be generalizable to the wider population of T2DM patients across Nigeria, particularly those in rural or underserved areas with limited access to specialized care. Second, the cross-sectional design limits causal inference between self-care practices and glycemic control. Third, by focusing on patients who were actively attending specialized clinics, the study population likely reflects individuals with relatively better health literacy, medication access, and care engagement, thus the findings may not reflect the realities of patients lacking regular access to healthcare. Future research should use community-based and longitudinal approaches to explore structural determinants and enhance representativeness across diverse settings.

## Conclusions

This study reveals that socio-demographic and clinical characteristics meaningfully shape self-care behaviors among patients with T2DM receiving specialized care in Nigeria. While adherence to foot care and medication use was relatively high, substantial gaps persist in dietary practices, physical activity, and blood glucose monitoring, which were all significantly associated with glycemic control. These findings, drawn from an urban clinical population predominantly of Hausa ethnicity, underscore the importance of interpreting results within the context of patients with better access to diabetes services and potentially higher health literacy. To improve diabetes outcomes, interventions should prioritize the behavioral domains with poorest adherence and be tailored to the cultural, ethnic, and socio-demographic profiles of different patient groups. At the same time, expanding care beyond specialized clinics through community-based strategies and structural reforms will be essential to reach underserved populations. Future studies should incorporate longitudinal designs and include rural and socioeconomically disadvantaged groups to better understand the temporal and structural determinants of self-care and glycemic outcomes across diverse Nigerian settings.

## Electronic supplementary material

Additional file 1.

## Declarations

### Availability of data and materials

The datasets generated and/or analyzed during the current study are available from the corresponding author on reasonable request.
