# Supplementary material for: Self-care behaviors and glycemic control in Nigerian patients with type 2 diabetes: a pilot cross-sectional study
Source: Glob Health Res Policy. 2025 Nov 11;10:60. doi: 10.1186/s41256-025-00427-9 (PMC12604327; doi:10.1186/s41256-025-00427-9)
Supplement: Supplementary file 1 — Additional file 1. [file 41256_2025_427_MOESM1_ESM.docx]

**Supplementary materials**

**Self-Care Behaviors and Glycemic Control in Nigerian Patients with Type 2 Diabetes: A Pilot Cross-Sectional Study**

Muhammad Abdullahi Idris^1,2,3✝^, Yanxin Bi^1✝^, Minmin Wang^1^, Ashiru Hamza Mohammad^4^, Yinzi Jin^1,5^*, Zhi-Jie Zheng^1,5^

1 Department of Global Health, School of Public Health, Peking University, Beijing, China

2 Department of Physiotherapy, Federal Medical Center, Gusau, Zamfara State, Nigeria

3 Department of Physiotherapy, Faculty of Basic Medical Sciences, Zamfara State University, Talata Mafara, Zamfara State, Nigeria

4 Department of Public Health, Godiya Disability Inclusion & Development Initiative (GDID), Dutse, Jigawa State, Nigeria

5 Institute for Global Health and Development, Peking University, Beijing, China

^✝^ These authors contributed equally to this study

* Correspondence to: Yinzi Jin, Ph.D., E-mail: yzjin@bjmu.edu.cn

**Supplementary Table 1** Differences in Fasting Blood Glucose Levels Across Socio-Demographic Variables.

| Variables | Normal | | Elevated | | χ^2^ | *P* |
| --- | --- | --- | --- | --- | --- | --- |
|  | n | % | n | % |  |  |
| **Gender** |  |  |  |  | 0.001 | 0.976 |
| Female | 76 | 54.29 | 64 | 45.71 |  |  |
| Male | 66 | 54.10 | 56 | 45.90 |  |  |
| **Age group** (years) |  |  |  |  | 38.692 | <0.001 |
| 18-40 | 35 | 89.74 | 4 | 10.26 |  |  |
| 41-60 | 49 | 37.12 | 83 | 62.88 |  |  |
| >60 | 58 | 63.74 | 33 | 36.26 |  |  |
| **Marital status** |  |  |  |  | 23.713 | <0.001 |
| Married | 63 | 41.45 | 89 | 58.55 |  |  |
| Single | 79 | 71.82 | 31 | 28.18 |  |  |
| **Duration of illness** (years) |  |  |  |  | 19.174 | <0.001 |
| 1-10 | 90 | 52.33 | 82 | 47.67 |  |  |
| 11-20 | 32 | 45.71 | 38 | 54.29 |  |  |
| >20 | 20 | 100.00 | 0 | 0.00 |  |  |
| Body Mass Index, **BMI** (kg/m^2^) |  |  |  |  | 4.687 | 0.196 |
| Under weight | 9 | 69.23 | 4 | 30.77 |  |  |
| Normal | 50 | 47.62 | 55 | 52.38 |  |  |
| Overweight | 41 | 62.12 | 25 | 37.88 |  |  |
| Obese | 42 | 53.85 | 36 | 46.15 |  |  |
| **Blood Pressure** (mmHg) |  |  |  |  | 1.857 | 0.173 |
| Normal | 111 | 56.63 | 85 | 43.37 |  |  |
| High Blood Pressure | 31 | 46.97 | 35 | 53.03 |  |  |
| **Religion** |  |  |  |  | 8.511 | 0.004 |
| Islam | 142 | 55.69 | 113 | 44.31 |  |  |
| Christianity | 0 | 0.00 | 7 | 100.00 |  |  |
| **Ethnicity** |  |  |  |  | 17.495 | <0.001 |
| Hausa | 112 | 59.89 | 75 | 40.11 |  |  |
| Yoruba | 13 | 27.08 | 35 | 72.92 |  |  |
| Igbo | 17 | 62.96 | 10 | 37.04 |  |  |

**Supplementary Table 2** Differences in scores on dimensions of SDSCA and MMAS-4 on demographic variables (Mean±SD)

|  | **Diet** | **Exercise** | **Self-daily blood glucose testing behaviors** | **Foot care** | **Medication adherence** | **Total score** |
| --- | --- | --- | --- | --- | --- | --- |
| **Gender** |  |  |  |  |  |  |
| Female | 21.26±0.58 | 5.03±0.41 | 0.84±0.17 | 12.20±0.40 | 3.68±0.04 | 43.01±11.18 |
| Male | 17.84±0.60 | 6.29±0.51 | 1.72±0.31 | 10.79±0.54 | 3.46±0.06 | 40.10±13.42 |
| *P* | <0.001 | 0.054 | 0.011 | 0.032 | 0.002 | 0.057 |
| **Age group** (years) |  |  |  |  |  |  |
| 18-40 | 23.13±5.38 | 4.87±5.11 | 0.21±0.62 | 12.56±4.30 | 3.67±0.48 | 44.44±11.89 |
| 41-60 | 19.39±7.08 | 6.26±5.39 | 2.10±3.71 | 10.82±5.89 | 3.56±0.56 | 42.13±13.51 |
| >60 | 18.59±7.00 | 5.00±5.14 | 0.46±0.69 | 12.15±4.76 | 3.56±0.67 | 39.77±10.45 |
| *P* | 0.002 | 0.138 | <0.001 | 0.079 | 0.583 | 0.116 |
| **Marital status** |  |  |  |  |  |  |
| Married | 18.22±7.20 | 5.03±4.98 | 1.83±3.51 | 11.24±5.59 | 3.49±0.65 | 39.82 |
| Single | 21.67±6.12 | 6.42±5.60 | 0.45±0.81 | 11.96±4.96 | 3.69±0.46 | 44.19 |
| *P* | <0.001 | 0.036 | <0.001 | 0.277 | 0.007 | 0.004 |
| **Duration of illness** (years) |  |  |  |  |  |  |
| 1-10 | 20.53±7.06 | 5.78±5.49 | 1.44±3.23 | 10.74±5.93 | 3.59±0.54 | 42.08±14.20 |
| 11-20 | 16.94±6.28 | 4.77±4.84 | 0.86±1.82 | 14.00±0.00 | 3.43±0.71 | 40.00±5.95 |
| >20 | 21.85±6.04 | 7.10±4.71 | 1.00±1.03 | 9.80±6.58 | 4.00±0.00 | 43.75±11.39 |
| *P* | <0.001 | 0.170 | 0.319 | <0.001 | <0.001 | 0.362 |
| **Body Mass Index, BMI** (kg/m^2^) |  |  |  |  |  |  |
| Under weight | 21.54±5.71 | 5.08±6.25 | 2.46±3.84 | 11.85±5.26 | 3.69±0.48 | 44.62±17.23 |
| Normal | 18.60±7.30 | 5.86±5.41 | 1.24±2.94 | 11.87±5.06 | 3.48±0.65 | 41.04±11.54 |
| Overweight | 21.33±6.56 | 5.89±5.18 | 0.98±2.66 | 11.67±5.26 | 3.73±0.51 | 43.61±11.42 |
| Obese | 19.40±6.82 | 5.14±5.09 | 1.28±2.51 | 10.95±5.82 | 3.56±0.55 | 40.33±13.14 |
| *P* | 0.062 | 0.763 | 0.389 | 0.701 | 0.046 | 0.315 |
| **Blood Pressure** (mmHg) |  |  |  |  |  |  |
| Normal | 19.82±7.30 | 5.20±5.23 | 1.05±2.06 | 11.00±5.76 | 3.61±0.61 | 40.67 |
| High | 19.24±5.91 | 6.85±5.28 | 1.85±4.29 | 13.15±3.37 | 3.47±0.50 | 44.56 |
| *P* | 0.564 | 0.028 | 0.044 | 0.004 | 0.088 | 0.027 |
| **Religion** |  |  |  |  |  |  |
| Islam | 19.61±6.92 | 5.61±5.34 | 1.28±2.83 | 11.58±5.30 | 3.57±0.59 | 41.66 |
| Christianity | 21.86±8.78 | 5.86±2.85 | 0.00±0.00 | 10.00±6.83 | 3.71±0.49 | 41.43 |
| *P* | 0.401 | 0.902 | 0.233 | 0.439 | 0.530 | 0.961 |
| **Ethnicity** |  |  |  |  |  |  |
| Hausa | 20.63±6.83 | 5.56±5.39 | 1.50±3.18 | 12.20±4.70 | 3.71±0.50 | 43.60±11.77 |
| Yoruba | 17.96±6.93 | 4.52±3.89 | 0.79±1.57 | 12.83±3.91 | 3.48±0.51 | 39.58±9.86 |
| Igbo | 16.07±6.40 | 7.93±6.13 | 0.33±0.48 | 4.67±6.73 | 2.81±0.68 | 31.81±15.00 |
| *P* | 0.001 | 0.026 | 0.059 | <0.001 | <0.001 | <0.001 |
| **Blood glucose level** (mmol/l) |  |  |  |  |  |  |
| Normal | 21.68±5.90 | 7.49±6.03 | 1.73±3.49 | 12.03±4.89 | 3.64±0.55 | 46.56 |
| Poor | 17.30±7.40 | 3.40±3.00 | 0.68±1.50 | 10.97±5.79 | 3.50±0.62 | 35.85 |
| *P* | <0.001 | <0.001 | 0.003 | 0.109 | 0.053 | <0.001 |

**SDSCA**: Summary of Diabetes Self-Care Activities; **MMAS-4**: Morisky’s Medication Adherence Scale-4.

**Supplementary Table 3** Association Between Self-Care Practices and Blood Glucose Levels.

| Variables | *OR* | *SE* | *Z* | *P* | *95% CI* | |
| --- | --- | --- | --- | --- | --- | --- |
|  |  |  |  |  | *Lower* | *Upper* |
| **Total score** | 0.83 | 0.03 | -5.88 | <0.001 | 0.78 | 0.88 |
| **Body Mass Index, BMI** (kg/m^2^) |  |  |  |  |  |  |
| Under weight |  |  |  |  |  |  |
| Normal | 5.80 | 5.32 | 1.92 | 0.055 | 0.96 | 34.98 |
| Overweight | 4.79 | 4.66 | 1.61 | 0.107 | 0.71 | 32.26 |
| Obese | 3.07 | 2.60 | 1.32 | 0.187 | 0.58 | 16.19 |
| **Blood Pressure** (mmHg) |  |  |  |  |  |  |
| Normal |  |  |  |  |  |  |
| High | 2.90 | 1.69 | 1.82 | 0.069 | 0.92 | 9.11 |
| **Gender** |  |  |  |  |  |  |
| Female |  |  |  |  |  |  |
| Male | 0.36 | 0.21 | -1.75 | 0.080 | 0.12 | 1.13 |
| **Age group** (years) |  |  |  |  |  |  |
| 18-40 |  |  |  |  |  |  |
| 41-60 | 652.31 | 755.83 | 5.59 | <0.001 | 67.32 | 6320.37 |
| >60 | 64.26 | 63.32 | 4.23 | <0.001 | 9.32 | 443.23 |
| **Marital status** |  |  |  |  |  |  |
| Married |  |  |  |  |  |  |
| Single | 0.07 | 0.05 | -3.65 | <0.001 | 0.02 | 0.28 |
| **Ethnicity** |  |  |  |  |  |  |
| Hausa |  |  |  |  |  |  |
| Yoruba | 130.80 | 133.73 | 4.77 | <0.001 | 17.63 | 970.27 |
| Igbo | 0.01 | 0.01 | -4.06 | <0.001 | 0.00 | 0.11 |
| **Duration of illness** (years) |  |  |  |  |  |  |
| 1-10 |  |  |  |  |  |  |
| 11-20 | 4.10 | 2.46 | 2.35 | 0.019 | 1.27 | 13.31 |
| >20 | 0.00 | 0.00 | -0.02 | 0.985 | 0.00 | . |

**Supplementary Table 4** Diagnostics for Multivariate Regression Models Across Five Self-Care Dimensions.

| Variables | Diet | Exercise | Self daily blood glucose testing behaviours | Foot care | Medication adherence |
| --- | --- | --- | --- | --- | --- |
| R² | 0.21 | 0.15 | 0.19 | 0.40 | 0.31 |
| Adjusted R² | 0.18 | 0.11 | 0.15 | 0.37 | 0.27 |
| F-statistic (p-value) | 5.67 (*P*<0.001) | 3.68 (*P*<0.001) | 4.79 (*P*<0.001) | 13.75 (*P*<0.001) | 9.18 (*P*<0.001) |
| Shapiro-Wilk Test (W) | 0.954 (*P*<0.001) | 0.922 (*P*<0.001) | 0.801 (*P*<0.001) | 0.920 (*P*<0.001) | 0.924 (*P*<0.001) |
| Root Mean Square Error (RMSE) | 6.32 | 4.99 | 2.59 | 4.24 | 0.50 |
| Mean VIF | 2.41 | | | | |

^*^*P*<0.05, ^**^*P*<0.010, ^***^*P*<0.001
